# Automated Machine-Learning Framework Integrating Histopathological and Radiological Information for Predicting IDH1 Mutation Status in Glioma

**DOI:** 10.3389/fbinf.2021.718697

**Published:** 2021-10-26

**Authors:** Dingqian Wang, Cuicui Liu, Xiuying Wang, Xuejun Liu, Chuanjin Lan, Peng Zhao, William C. Cho, Manuel B. Graeber, Yingchao Liu

**Affiliations:** ^1^ School of Computer Science, The University of Sydney, Sydney, NSW, Australia; ^2^ Department of Neurology, Shandong Provincial Hospital Affiliated to Shandong First Medical University, Jinan, China; ^3^ Department of Radiology, Hospital Affiliated to Qingdao University, Qingdao, China; ^4^ Department of Neurosurgery, Shandong Provincial Hospital Affiliated to Shandong First Medical University, Jinan, China; ^5^ Department of Neurosurgery, Shandong Provincial Hospital Affiliated to Shandong University, Jinan, China; ^6^ Department of Clinical Oncology, Queen Elizabeth Hospital, Kowloon, Hong Kong, SAR China; ^7^ Ken Parker Brain Tumor Research Laboratories, Brain and Mind Centre, Faculty of Medicine and Health, The University of Sydney, Sydney, NSW, Australia

**Keywords:** digital histological slides, isocitrate dehydrogenase 1 mutations, machine-learning, magnetic resonance imaging, multimodal integration, imaging information analysis

## Abstract

Diffuse gliomas are the most common malignant primary brain tumors. Identification of isocitrate dehydrogenase 1 (IDH1) mutations aids the diagnostic classification of these tumors and the prediction of their clinical outcomes. While histology continues to play a key role in frozen section diagnosis, as a diagnostic reference and as a method for monitoring disease progression, recent research has demonstrated the ability of multi-parametric magnetic resonance imaging (MRI) sequences for predicting IDH genotypes. In this paper, we aim to improve the prediction accuracy of IDH1 genotypes by integrating multi-modal imaging information from digitized histopathological data derived from routine histological slide scans and the MRI sequences including T1-contrast (T1) and Fluid-attenuated inversion recovery imaging (T2-FLAIR). In this research, we have established an automated framework to process, analyze and integrate the histopathological and radiological information from high-resolution pathology slides and multi-sequence MRI scans. Our machine-learning framework comprehensively computed multi-level information including molecular level, cellular level, and texture level information to reflect predictive IDH genotypes. Firstly, an automated pre-processing was developed to select the regions of interest (ROIs) from pathology slides. Secondly, to interactively fuse the multimodal complementary information, comprehensive feature information was extracted from the pathology ROIs and segmented tumor regions (enhanced tumor, edema and non-enhanced tumor) from MRI sequences. Thirdly, a Random Forest (RF)-based algorithm was employed to identify and quantitatively characterize histopathological and radiological imaging origins, respectively. Finally, we integrated multi-modal imaging features with a machine-learning algorithm and tested the performance of the framework for IDH1 genotyping, we also provided visual and statistical explanation to support the understanding on prediction outcomes. The training and testing experiments on 217 pathologically verified IDH1 genotyped glioma cases from multi-resource validated that our fully automated machine-learning model predicted IDH1 genotypes with greater accuracy and reliability than models that were based on radiological imaging data only. The accuracy of IDH1 genotype prediction was 0.90 compared to 0.82 for radiomic result. Thus, the integration of multi-parametric imaging features for automated analysis of cross-modal biomedical data improved the prediction accuracy of glioma IDH1 genotypes.

## Introduction

The current WHO classification of CNS tumors not only considers histopathological phenotypes but also molecular genetic parameters, e.g., DNA methylome profiling (Louis et al., 2021; Lopes 2017; Chang et al., 2018; Lee et al., 2019). IDH mutations in glioma have been found to be associated with better outcomes and are therefore of great relevance in the clinical assessment of glioma patients (Louis et al., 2016b). Recently, some attempts have been made to use radiological images for the pre-surgical prediction of IDH1 genotypes (Gillies et al., 2015; Kesler et al., 2019; Lee et al., 2019; Tatekawa et al., 2021).

Pathological and radiological imaging results are increasingly available in digitized format (Nance et al., 2013; Farahani and Pantanowitz 2015; Griffin and Treanor 2017). It has become apparent that fully utilizing the data of digital radiology and pathology images through machine-learning can facilitate the identification of biomarkers that reflect information on the basic biology and physiology of various malignancies (Deo 2015; Gillies et al., 2016). Although tumor diagnoses increasingly consider molecular genetics markers, histology continues to play a key role in frozen section diagnosis, as a diagnostic reference and as a method for monitoring disease progression. In addition, compared to the indirect visualization of disease phenotypes by means of imaging, histology provides direct information at high resolution (Kinjo et al., 2008; Missbach-Guentner et al., 2018; Vågberg et al., 2018; Rathore et al., 2020). Competing with the computer-aid image technique used in radiology and pathology, clinical practice demands professional knowledge and long-term training to obtain useful information from the image with the naked eye for diagnosis and evaluation (Harezlak and Kasprowski 2018).

Computerized image analysis can reduce subjective inter-observer bias that is known to limit all human observation including in histopathology (Emblem et al., 2014; Zhang et al., 2016; Choi et al., 2019). Machine-learning algorithms are already widely used in glioma research, and most are based on the analysis of features extracted from MRIs (Ellingson et al., 2011; Zacharaki et al., 2012; Emblem et al., 2014; Macyszyn et al., 2015). Zhou et al. (2017) have recently demonstrated the ability to predict IDH genotypes in cases of primary grade II and III glioma using clinical and pathological variables and textual features extracted from regions of interest (ROI) in four sequences of MRI, including T1W, T2W, T1CE and T2-FLAIR, achieving an accuracy of 0.86. Compared to the results obtained by Zhou and colleagues, Eichinger et al. (2017) were able to increase the accuracy of IDH genotyping to 92% by designing an algorithm based on feature extraction of local binary pattern, which represent texture features extracted from multimodal MRI data. In addition to traditional machine-learning techniques, the method employed by Xing et al. (2017) classifies IDH mutations and IDH wild type (IDH-wt) using conventional machine-learning algorithms in order to extract deep features from four sequences of MRI (T1W, T2W, T1CE, T2-FLAIR). Zhang et al. (2016) aimed to distinguish the presence of an IDH mutation and IDH-wt in primary grade III and IV gliomas by means of additional features (intensity, texture and shape features) extracted from multimodality MRIs (T1, T1CE, T2, T2-FLAIR and DWI), achieving an accuracy of 0.883 using the Random Forest algorithm.

Recently, the improvements in deep learning is capable to overcome the previous challenges by learning high-dimensional representations of imaging data. Novel, fully automated postprocessing analyses of standard and advanced MR images are clearly rapidly approaching. These fully automated analyses are especially appealing because they provide unbiased evaluations independent of operator, training or experience. Fully automated postprocessing with deep learning analyses of standard and advanced MR images have achieved high accuracy even at 92.8% accuracy, 93.1% specificity, and 92.6% sensitivity (Choi et al., 2019). Although they can be very powerful for the prediction of IDH for glioma, deep CNN models are vulnerable to overfitting to their given training dataset and inherent difficult for interpretation which is the most crucial for decision support system. Comprehensive understanding of the mechanism of deep and machine-learning is necessary, however, to best develop and then apply these algorithms to clinical practice we need to avoid their potential pitfalls. It is unlikely to replace tissue sampling for now; therefore, the continued improvement in model performance and consistency across diverse imaging modality brings us closer to the precise molecular diagnosis (Gutman and Young 2021).

In this study, we introduce an improved approach to IDH prediction, which integrates radiological and histopathological data analyses in a single combined framework. Radiological data analysis in this context refers to the extraction and analysis of high-throughput features from tomographic images (MR images) whereas histopathological data analysis refers to the features extraction and analysis from whole slide images. We envision that this model could set a pathway for the non-invasive evaluation of IDH mutation in gliomas and may provide a quantitative result analysis for the researchers. Compared with deep learning-based method, we aim to provide doctors with an intuitive, interpretable, and cost-effective mechanism through machine-learning based method to support the decision on IDH status prediction.

## Materials and Methods

### Patient Enrolment

The imaging data of 217 subjects that had been diagnosed with glioma were collected from two different sources. 126 cases were from Shandong Provincial Hospital that is affiliated with Shandong University, comprising 41 histological grade III cases and 85 histological grade IV cases. The remaining 91 cases were retrieved from The Cancer Imaging Archive (TCIA), comprising 25 histological grade III cases and 66 histological grade IV cases (Table 1). The criteria for image acquisition in this study are as follows: I) available histology, age at diagnosis, sex, and IDH status; II) MR imaging data, including post-contrast T1-weighted images (T1CE), and T2-FLAIR, and III) histopathological images.

**TABLE 1:** Patient characterizes.

	Shandong Provincial Hospital	TCGA^a^
Grade III (*n*; %)	41	25
IDH-mutated in Grade III (*n*; % column)	20, 48.9%	17, 68%
Grade IV (*n*; %)	85	66
IDH-mutated in Grade IV (*n*; % column)	20; 23.5%	12, 18.2%
Age (years; mean; range)	49; [5, 79]	53; [18, 81]
Sex (n male; % column)	55; 43.7%	55; 60.4%

a TCGA, The Cancer Genome Atlas.

### Dataset

#### Histopathological Images

Shandong provincial hospital’s cohort: cases were diagnosed according to WHO criteria (Louis et al., 2021). Paraffin-embedded tissue samples were cut into 3 μm thick slides and stained with H&E stain for all patients in this cohort. All H&E stained images were scanned on a Leica SCN400 slide scanner (Leica Biosystems, Nussloch, Germany) with multi-resolution varying from 20× to 40× for analysis.

Genomic DNA was isolated from formalin-fixed paraffin-embedded glioma tissues. DNA was extracted using the QIAamp DNA Micro kit (Qiagen GmbH, Hilden, Germany) as previously described (Perizzolo et al., 2012).

Expression of IDH-R132H mutant was firstly analyzed by IHC as previously described (Reyes-Botero et al., 2014). For IDH R132H–negative tumors, multiple-gene Sanger sequencing was performed to identify alternative IDH mutations (Sanson et al., 2009). IDH status was defined according to the absence of IDH-R132H immunopositivity and/or mutations in IDH1 and IDH2 genes identified by sequencing.

The Cancer Genome Atlas (TCGA) cohort: Digital pathology slides of diagnosed diffuse gliomas were downloaded from TCGA Data Portal (http://cancergenome.nih.gov.) including information on IDH status, and the corresponding MRI images were acquired from the Cancer Imaging Archive (TCIA) Data Portal (https://www.cancerimagingarchive.net).

#### Multimodal MR Images

All patients were imaged in the supine position with a 3.0-T MRI machine (Magnetom, Skyra; Siemens Healthcare, Erlangen, Germany) using a transmit/receive quadrature 20-channel head-and-neck coil. The imaging protocol was the same for all patients.

T1-contrast: TR, 1820 ms; TE, 13 ms; slice number, 19; FOV, 230 mm; slice thickness, 5 mm; distance factor, 30%; FA, 150 deg; inversion time (TI), 825 ms; voxel size, 0.4 × 0.4 × 5.0 mm; accelerate factor, 2; bandwidth, 260 Hz/Px; echo spacing, 13 ms.

Fluid-attenuated inversion recovery imaging (T2-FLAIR): TR, 8,000 ms, TE: 81 ms, slice number: 19, FOV, 220 mm; slice thickness, 5 mm; distance factor, 30%; FA, 150 deg; inversion time (TI), 2,370 ms; voxel size, 0.7 mm × 0.7 mm × 5.0 mm; accelerate factor, 2; bandwidth, 289 Hz/Px; echo spacing, 9.02 ms.

All MRI sequences of each patient from our own datasets and from TCIA have the same imaging scale, position, slice anatomy and slice thickness.

## Computer Analysis

**FIGURE 1:**
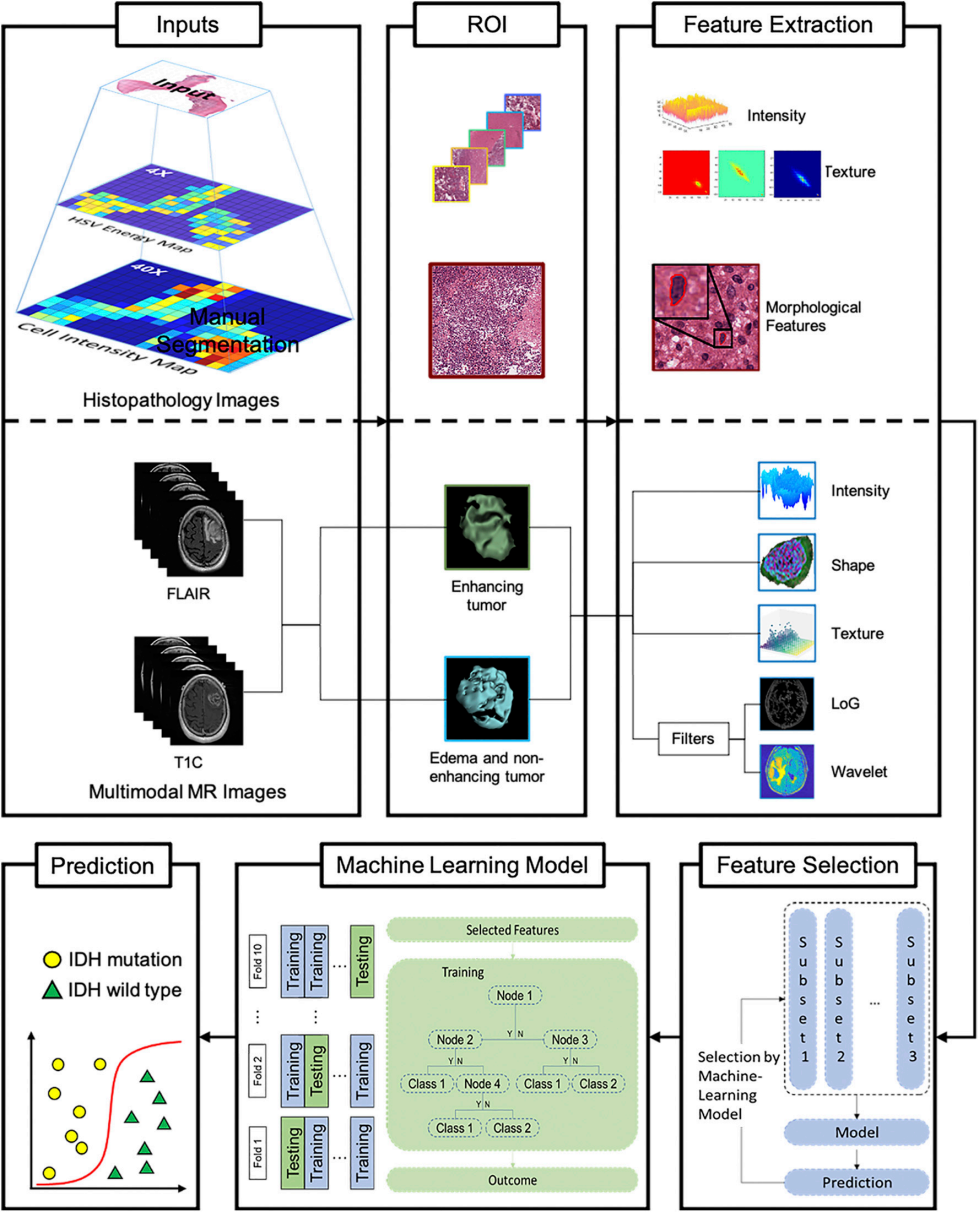
Machine-learning framework for automated prediction of IDH glioblastoma genotypes. Histopathology and multimodal MR images are used as input (left column). Representative regions of interest (ROIs) are extracted (middle column) followed by histopathological and radiomics feature selection (right column). Subsequently, a Random Forest model-based Recursive Feature Elimination (RF-RFE)-algorithm is applied to select relevant while reducing redundant features. Following 10-fold cross-validation, the automated machine-learning model for glioma IDH genotype prediction is established. Abbreviations: GCLM, Grey Level Co-occurrence Matrix features; GLRLM, Grey Level Length Matrix features; GLSZM, Grey Level Size Zone Matrix features; NGTDM, Neighboring Gray Tone Difference Matrix features; GLDM, Grey level Dependence Matrix features, mass of features, which may have redundant information, then selected and processed to improve the predictive power of the machine-learning model.

An automated framework was designed to predict IDH genotype, consisting of the following steps, which were carried out in sequence: I) automated image pre-processing to select the regions of interest (ROIs), II) feature extraction, III) feature selection, and IV) automated IDH genotype prediction and results interpretation (Figure 1).

### Automatic Extraction of Region of Interest

Our computational algorithm used for the analysis of histological images approaches the region of interest at two different levels. First, one tile with the highest cell density (5,120 * 5,120) (Sertel et al., 2009; Mobadersany et al., 2018) is extracted employing the watershed nuclei detection algorithm (Al-Kofahi et al., 2010; Kumar et al., 2017; Wang et al., 2018). Then, based on Hue, Saturation, and Value of Brightness (HSV channel), five tiles representing the whole image (Al-Kofahi et al., 2010; Kumar et al., 2017) at 40X resolution are identified. Third, based on the HSV channel, five tiles representing the entire image at 4X resolution are identified.

For the analysis of radiological images, we have segmented edema and non-enhanced tumors from T2-FLAIR image. In addition, T1CE images were used for enhancing tumor volume segmentation. The lesions were separated into three parts, enhancing tumor, tumor necrosis and peritumoral edema. The process of tumor segmentation was performed manually using the ITK-SNAP software (version 3.6.0; www.itksnap.org). First, all MRI sequences were retrieved from the Picture Archiving and Communication System (PACS). Then we applied N4 bias field correction to remove the presence of low frequency intensity non-uniformity. Inter-modality co-registration with different 2D MRI sequences was achieved by means of ITK-SNAP. Using this method, ROIs of enhancing tumor were delineated on post-contrast T1WI images by a semi-automatic method, in which only the enhancing area was selected. Tumor necrosis was defined as the non-enhancing area within enhancing area on post-contrast T1WI. ROIs of peritumoral edema were delineated on T2-FLAIR, which was defined as the high-signal region beyond the enhancing area. The process was performed by a consultant neuro-radiologist. Finally, the ROIs were registered on each slice of each 2D MRI sequence.

### Feature Extraction

In this step, we extracted quantitative features from histopathology images and MRIs. In case of the histopathology images, we extracted two types, visual features and sub-visual features, at two different resolutions. The visual features quantitatively describe the morphology of nuclei such as the mean area occupied and the pattern of staining. Sub-visual features are derived from a high-throughput intensity and texture matrix, which reflects the intensity distribution at the single pixel level.

In the case of MRIs, we obtained shape features from the volume of interest (VOI) reflecting tumor area and volume. Subsequently, we extracted first-order, second-order and high order features from the ROIs. The shape-based features describe the three-dimensional (3D) properties of the tumor, such as tumor volume, sphericity, and 3D diameter. First-order statistical features reflect the distribution of voxel intensities within the tumor area, including energy and entropy. Second-order statistical features were obtained from the relationships between adjacent voxels (Balagurunathan et al., 2014) to describe the second-order joint probability function of the tumor region as a gray-level co-occurrence matrix (GLCM) and Gray-level run-length matrix (GLRLM), respectively, which reflect intra-tumoral heterogeneity. High-order features were calculated with the help of different filters such as the wavelet transform.

### Feature Selection

Although a large number of image features can be used to construct a model that fully reflects the characteristics of gliomas, removing redundant information can improve the efficacy of the model for glioma genotyping (Guyon and Elisseeff 2003). In order to reduce the amount of redundant information inherent to quantitative features, we built a Random Forest algorithm enhanced by a recursive feature elimination (RF-RFE) procedure in order to identify the relevant and important characteristics before implementation in a classification model all (Saeys et al., 2007). As shown in Figure 1, the feature with the lowest importance for classification calculated by the algorithm will be eliminated.

### Modeling and Validation

We are proposing a binary classification model to differentiate patients with an IDH mutation from wild type high-grade gliomas (HGGs) based on clinical features (age and sex), digital histopathological image features and MRI Radiomics features.

The Random Forest algorithm employed in this study is widely used in medical imaging analysis. The corresponding model is able to accommodate a very large set of features. All machine-learning methods were implemented with the Statistics and Machine-Learning package on Python 3.6.

As discussed by Guyon and Elisseeff (2003), although a large number of image features can be used to construct a model to better reflects the characteristics of gliomas, the model may face over-fitting problems, and therefore redundant information needs to be carefully removed to improve the efficacy of the model for genotyping gliomas. In order to ensure the stability and efficiency of the selected features, the 10-Fold cross-validation is nested with the RF training model to select a valuable feature set. The random forest algorithm enhanced by the recursive feature elimination (RF-RFE) process is used to identify relevant and important features before all implementations in the classification model (Saeys et al., 2007).

## Results

### Feature Extraction and Selection

We extracted a total of 22 morphological features, which were identified in the glioma cases studied (Supplementary Material I. Extraction of histopathologic features), including nuclear shape and staining intensity (Figure 1). In addition, we extracted 171 sub-visual features (Wang et al., 2018) from the high resolution digital histopathology images, including intensity features and GLCM features.

As for results of IDH status prediction for HGGs, the histopathological features extracted from histopathology images, which reached an accuracy of 0.81 ± 0.03 with 10-fold cross validation. Regarding multimodal MRIs (Supplementary Material II. Radiomics Features Extraction), 1,132 features were extracted from the individual patients’ different MR image sequences, including 234 first order features, 14 shape-based features, 286 grey level co-occurrence matrix features, 208 grey level run length matrix features, 208 grey level size zone matrix features, and 182 grey level dependence matrix features (Figure 1). The area under the curve (AUC) for features extracted from different histopathological grade of tumors was 0.90 ± 0.09.

### Comparison of Performances When Using Different Modalities and Feature Types

**FIGURE 2:**
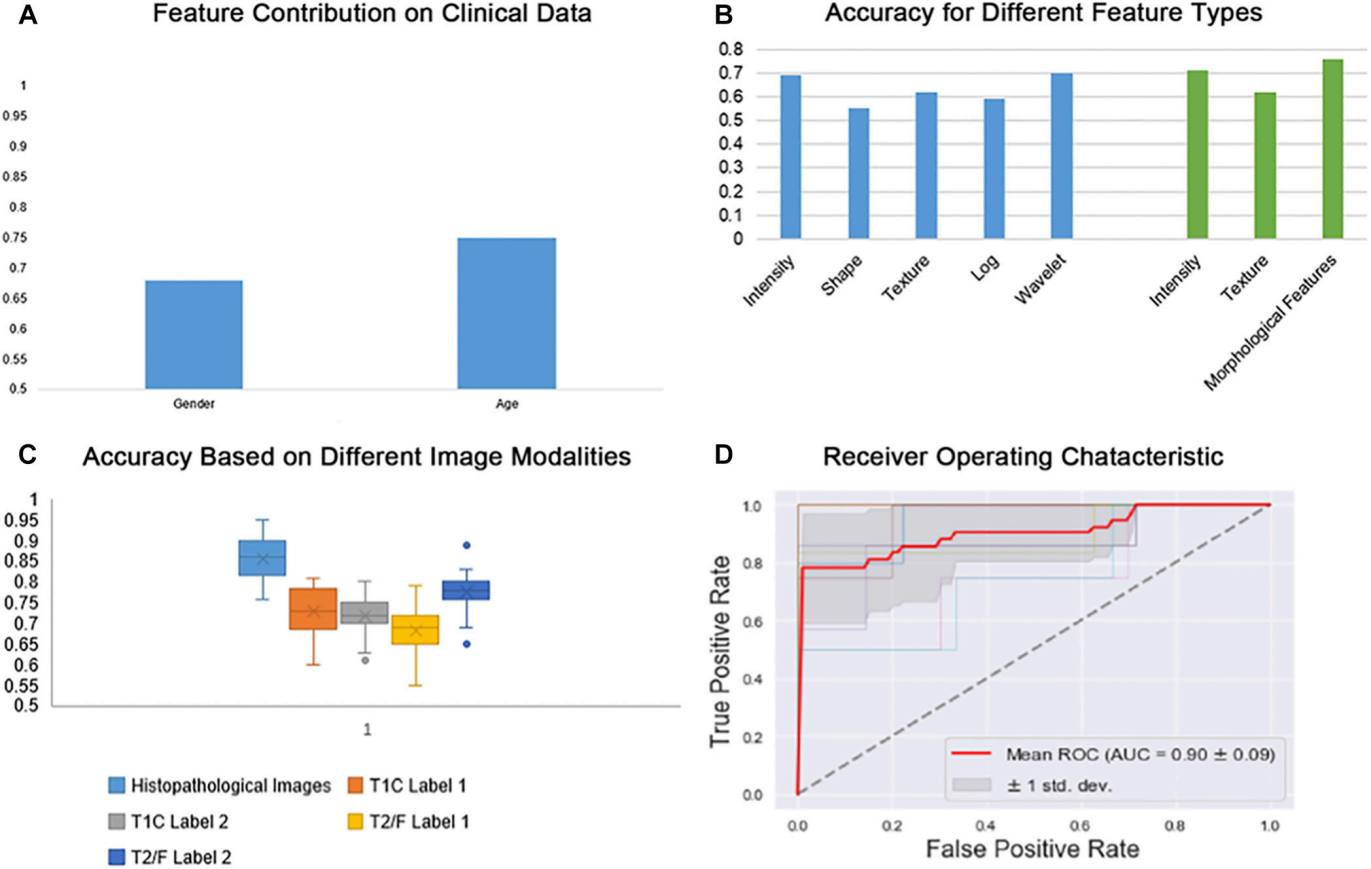
Random forest classifier scores for IDH genotype prediction. **(A)** Prediction results based on Clinical data (age and gender); **(B)** Prediction results based on different feature types; **(C)** Prediction results based on different image modalities; **(D)** Receiver Operating Characteristic (ROC) for IDH genotype prediction across multi-parametric medical images.

In order to assess the differential relevance of the modalities tested (T1CE, FLAIR and digital pathology images) in the prediction of IDH genotype, a Random Forest machine-learning model with 10-fold cross-validation was established. In general, scans of histopathological images yielded more accurate results in the IDH genotype prediction than other image types (Figure 2C). Considering quantitative features obtained from the different modalities, our morphologically defined visual features also showed high accuracy and stability (Figure 2B). With the multiparameter imaging features minded from different modalities images, our quantitative and objective analysis platform achieved high diagnostic accuracy (0.90 ± 0.05). On the other hand, the mined multiparametric features were achieved different accuracy in corresponding image modality, including 0.86 (±0.03) in the Digital Histopathological Images, 0.73 (±0.06) in T1CE (edema and non-enhanced tumor), 0.72 (±0.04) in T1CE (enhanced tumor), 0.68 (±0.05) in T2-FLAIR (edema and non-enhanced tumor) and 0.78 (±0.04) in T2-FLAIR (enhanced tumor).

For IDH genotype prediction, optimal features were selected from the different modalities of medical images, including seven features from the digital histopathological images, four features form the T1CE images and five from the T2-FLAIR images. As shown in Figure 2B, GLRLM, Shape-based and GLCM features had the greatest power in predicting glioma IDH status. Age, counts of nuclei and first-order features were the most important factors that contributed to this result. Top-performers within different groups of image features contributed to IDH status prediction as summarized in Table 2.

**TABLE 2:** TOP-performing features in IDH status prediction by means of univariate analysis.

Types	Mask	Feature name	Feature description	Accuracy
Clinical	N/A	Age	Age at diagnose	0.74
Intensity	T1C-edema	Uniformity	Formula	0.69
			$F_{u}=\;\overset{N_{g}}{\underset{i=1}{\sum }}p{\left(i\right)}^{2}$	
			Where ${\text{p}}_{\left(\text{i}\right)}$ refers to the features calculated form ${\text{N}}_{\text{g}}$ discrete pixel levels	
			Measuring the sum of the square of image VOI pixel value	
Shape	FLAIR-edema	Flatness	Formula	0.67
			$F_{flatness}=\;\frac{{\text{λ}}_{least}}{{\text{λ}}_{major}}$	
			Where ${\text{λ}}_{major}$ and ${\text{λ}}_{least}$ refer to the length of the maximum and minimum principal component axes, respectively	
			Measuring the relationship between the largest and smallest principal components in the VOI shape	
Texture	T1C-tumor	Wavelet-LLL_glrlm_LRLGLE	Formula	0.72
			$F_{L}=\;\frac{\sum_{i=1}^{N_{g}}\sum_{j=1}^{N_{r}}\frac{P\left(i,j|\theta \right)j^{2}}{i^{2}}}{N_{r}\left(\theta \right)}$	
			Where $N_{g}$ refers to the gray level distribution within the VOI, $N_{r}$ refers to the maximal length within the VOI, P(i,j|θ) refers to the run length matrix for an arbitrary direction θ , $N_{r}\left(\theta \right)$ is the number of runs in the image along θ	
			This feature quantitative describes the joint distribution of long-run lengths with lower gray level values after a wavelet filter	
Wavelet	T1C-tumor	wavelet-HHH-glcm-MP	Formula	0.69
			$F_{MP}=\text{max}\left(p\left(i,j\right)\right)$	
			Where p(i,j) is the normalized co-occurrence matrix	
			Quantify the occurrences of the most predominant pair of neighboring intensity values through a Gray Level Co-occurrence Matrix after an image filter by a high-frequency wavelet.	
LoG	FLAIR-tumor	Log-glszm- SALGLE	Formula	0.65
			$F_{SALGLE}=\;\frac{\sum_{i=1}^{N_{g}}\sum_{j=1}^{N_{s}}\frac{P\left(i,j\right)}{i^{2}j^{2}}}{N_{z}}$	
			Where $N_{g}$ refers to the distribution values within the VOI, $N_{s}$ refers to the zone sizes quantity within the VOI., $N_{z}$ refers to refers to the zones quantity within the VOI, and P(i,j) is the size zone matrix	
			Quantify the proportion in the mask of VOI by quantify the Gray Level Size Zone joint distribution of smaller size zones with lower gray level values after the LoG filter	
Morphology	Tile	Cell counts	Quantitative describes the cell intensity in the ROI.	0.78

MP, Maximum Probability; SALGLE, Small Area Low Gray Level Emphasis; LRLGLE, Long Run Low Gray Level Emphasis; HHH, high, high and high frequency.

The accuracy of IDH status prediction was as high as 0.88 ± 0.03 when multi-parametric features were extracted from different histopathological and radiomics images through our implementation of the Random Forest algorithm. Table 3 shows the features that play important roles in our classification model.

**TABLE 3:** Prediction of IDH genotype based on high grade gliomas.

Image modalities	Accuracy
Digital Histopathological Images	0.86 (± 0.03)
T1CE (edema and non-enhanced tumor)	0.73 (± 0.06)
T1CE (enhanced tumor)	0.72 (± 0.04)
T2-FLAIR (edema and non-enhanced tumor)	0.68 (± 0.05)
T2-FLAIR (enhanced tumor)	0.78 (± 0.04)
Multi-modal Image Data	0.90 (± 0.05)

### Quantitative Isocitrate Dehydrogenase Status Prediction and Results Interpretation

**FIGURE 3:**
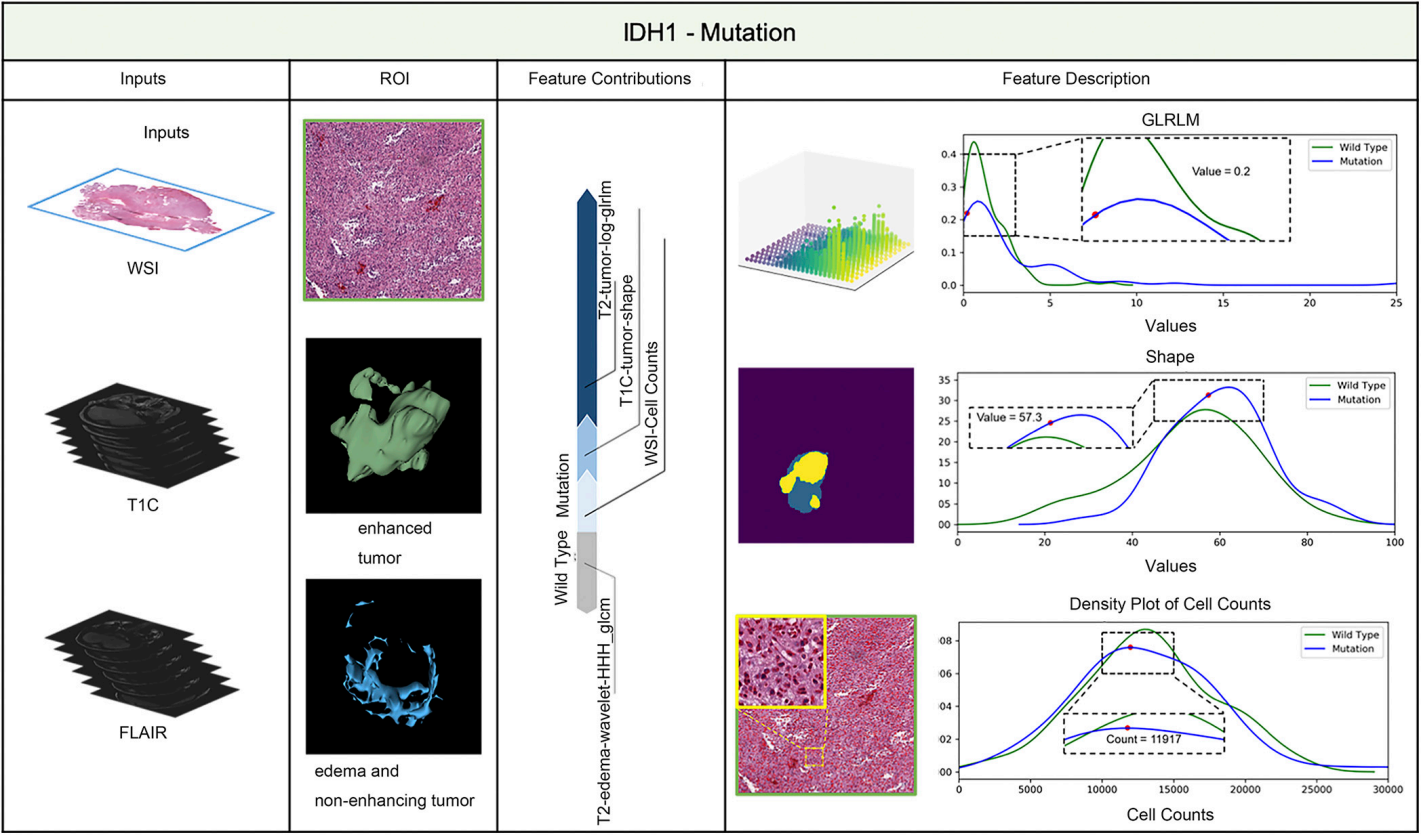
IDH status prediction result explanation of a representative case with the LIME algorithm for the RF model (IDH-Wild type case).

**FIGURE 4:**
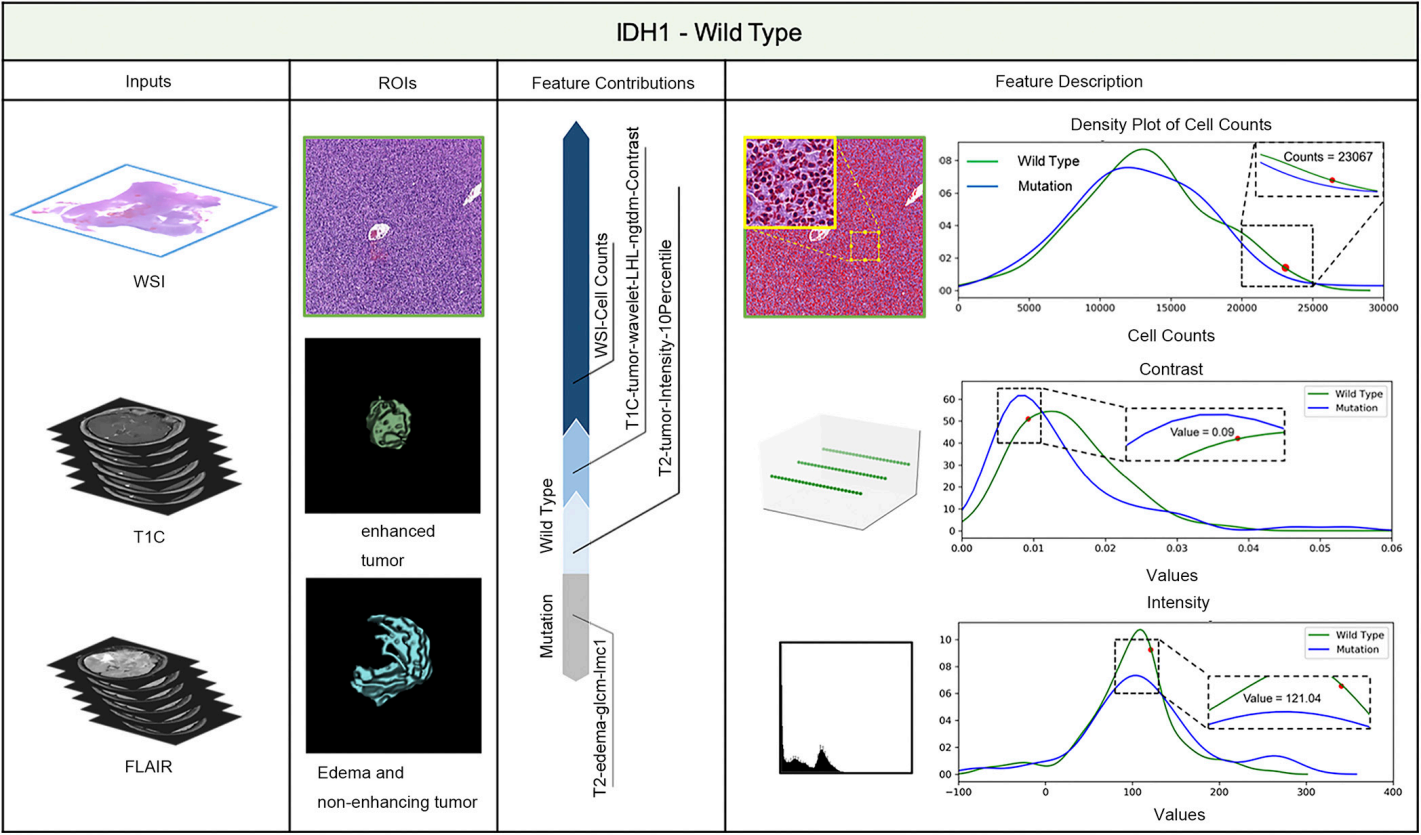
IDH status prediction result explanation of a representative case with the LIME algorithm for the RF model (IDH-Mutation case).

LIME (Local Interpretable Model-agnostic Explanations) is a tool for facilitating local model interpretability. The technique perturbs the input data to understand how the predictions are affected. Figures 3, 4 illustrate two representative cases from visual analysis and the machine-learning model. The first case is an IDH-wt patient (Figure 3), who is 43 years old (age at diagnosis), female with a histopathological grade IV glioma. The second one is a patient with an IDH mutation (Figure 4), who is 22 years old (age at diagnosis), male with histopathological grade III glioma.

Machine-learning models taking into account the extracted features’ different contributions, then quantitatively predict the results by combining these different features according to their corresponding contributions. During model training, the LIME model can generate weighted coefficients to illustrate the contributions made by different features. The predictive ability of LIME algorithm made the most important contribution to achieve a higher weight value. Positive weights reflect the increase in the corresponding features may make a positive contribution to the IDH status prediction. In contrast, negative weights would have negative predictive power. As shown in Figures 3, 4, the feature contribution for IDH genotyping for two representative cases have been listed, which are derived from the LIME model to obtain the linear combination of feature values and weights.

## Discussion and Conclusion

### Discussion

Determination of IDH status has become a standard for glioma diagnosis as it helps to guide clinical decision-making. In this study, we have developed a Random Forest algorithm-based genotype classifier that allows the prediction of IDH mutation status in glioma patients from pre-surgical MRI scans (Zhang et al., 2016) with improved accuracy. The Random Forest algorithm-based genotype classifier aims to employ the machine-learning algorithm to do the IDH genotype and a stable and efficient prediction result of IDH genotype. In this situation, the Random Forest machine-learning model with 10-fold cross-validation was implement into this experiment. To be more specific, we apply the RF algorithm into the experiment due to the following advantages: I) in specific experiments, training can be highly parallelized and run efficiently on large data sets; II) since the partition features of decision tree nodes can be selected randomly, which leads to the input samples with high-dimensional features can be processed without dimensionality reduction; III) the algorithm is able to calculate the importance of each feature to the prediction result and IV) due to the adoption of random sampling and random feature selection, the model has small variance at the training location and strong induction ability. We adopt the RF into the experiment, due to the advantages the model have which match the height of our datasets.

In order to improve the accuracy of IDH phenotype prediction, visual and sub-visual features extracted from digital histopathological images and quantitative radiomics feature extracted from different multimodality MRIs were implemented into our Random-Forest-Recursive Feature Elimination (RF-RFE) feature selection model to identify optimal criteria for further analysis. In this task, the “visual features” refer to not only the basic features including the color and appearance of nuclear staining, but also non-basic features including different directions. On the other hand, the “sub-visual features” allude to the computerized high-throughput first-order and second-order features, which includes intensity and texture information. In this experiment, features were extracted from different conduits of the H&E images, which aims to improve the prediction accuracy of the IDH phenotype.

Our novel integrated approach, which combined multi-parametric biomedical imaging features, was found to be a more accurate predictor of IDH genotype than either radiomics or histopathological feature recognition alone. Multi-parametric biomedical imaging characterizes tumor properties at different biological levels, it meets the need to understand correlations between image features, genomics, and clinical outcomes.

Specifically, the IDH predictive performance of histopathological images was found to be superior to T2-FLAIR and T1CE (0.86 vs. 0.71, 0.75). Among the leading histomorphometrical features, the mean cell area and the mean cell axis were most significant. These top identified features mirror the fact that gliomas with an IDH mutation have a more coherent nuclear architecture, i.e., they are morphologically less atypical than IDH wild type, which is associated with a higher risk of recurrence.

Our IDH genotype prediction achieved high accuracy for mainly two reasons: First, we integrated MRIs, digital histopathological images and clinical information for IDH prediction. Second, we used the selected features to significantly reduce the number of parameters in the model to avoid overfitting while making our model more robust. To the best of our knowledge, this is the first study to integrate MRI and digital pathology images in a computerized model for predicting IDH genotype. It is worth noting that T1CE and T2-FLAIR images conferred a higher predictive value than other MR sequences.

### Conclusion

In conclusion, our work is a step towards a more effective use of radiomic and histopathological data. It should be particularly helpful for retrospective studies on gliomas where imaging results are available but also to point of care that do not have timely access to a molecular genetics laboratory. To sum up, our results i) demonstrate that machine-learning is capable of indirectly identifying genetic information within structural MR images and histopathological datasets, ii) suggest a complementary method for the IDH genotyping of gliomas suitable for patient screening, and iii) demonstrate the potential for algorithmic tools to support clinical decision-making. Taken together, it is expected that the integration of multimodal biomedical data analysis will become more popular in oncology research and practice as technology evolves, with significant potential for the future clinical management of brain tumor patients.

## Data Availability

The raw data supporting the conclusions of this article will be made available by the authors, without undue reservation.

## Abbreviations

TCGA, The Cancer Genome Atlas; TCIA, The Cancer Imaging Archive; IDH, Isocitrate dehydrogenase; IDH-wt, Isocitrate dehydrogenase wild type; IDH-m, Isocitrate dehydrogenase mutation; MRI, Magnetic Resonance Imaging; T1C, T1-weighted post-Gd contras; FLAIR, Fluid-attenuated inversion recovery; GLCM, Grey Level Co-occurrence Matrix; GLRLM, Grey Level Length Matrix; GLSZM, Grey Level Size Zone Matrix; GLDM, Grey level Dependence Matrix; LoG, Laplacian of Gaussian; ROI, Region of Interest; VOI, Volume of Interest; LIME, Local Interpretable Model-agnostic Explanations; ROC, Receiver Operating Characteristic; Label 1, the area of edema and non-enhancing tumor; Label 2 the area of enhanced tumor.
