# Supplementary material for: Automated Machine-Learning Framework Integrating Histopathological and Radiological Information for Predicting IDH1 Mutation Status in Glioma
Source: Front Bioinform. 2021 Oct 26;1:718697. doi: 10.3389/fbinf.2021.718697 (PMC9581043; doi:10.3389/fbinf.2021.718697)
Supplement: Supplementary file 1 [file DataSheet1.PDF]

Supplementary Materials

1 Histopathologic Feature Extraction

As shown in Supplementary Table 1, we have extracted two type of features, visual features and sub-visual features, at two different resolutions.

Supplementary Table 1. Histopathologic Features.

Visual Features	Sub-visual Features	
	**Intensity**	**Texture**
Counts	Energy	Autocorrelation
Area	Total Energy	Cluster Prominence
Perimeter	Entropy	Cluster Tendency
Circularity	10Percentile	Contrast
Solidity	90Percentile	Correlation
Min axis	Interquartile Range	Difference Average
Max axis	Kurtosis	Difference Entropy
Hematoxylin mean	Maximum	Difference Variance
Hematoxylin std dev	Minimum	Id
Hematoxylin min	Mean	Idm
Hematoxylin max	MAD	Idmn
Hematoxylin range	Median	Idn
	Range	Imc1
	rMAD	Imc2
	RMS	Inverse Variance
	Standard Deviation	Joint Average
	Uniformity	Joint Energy
	Variance	Joint Entropy
	Skewness	Max Probability
		Sum Entropy
		Sum Squares
		GLNU
		GLNUN
		LRE
		LRHGLE
		LRLGLE
		LRLGLE
		LGLRE
		Run Entropy
		RLNU
		RLNUN
		Run Percentage
		Run Variance
		SRE
		SRHGE
		SRLGE
		GLV

2 Radiomics Features Extraction

As shown in Supplementary Table 2, radiomics features extracted from 2 modalities of the 2 types of region of interests of the MRIs have been listed.

Supplementary Table 2. Radiomics Features.

**Shape (13)**	**Intensity (19)**	**GLRLM (16)**	**GLCM (24)**	**GLSZM (16)**	**NGTDM (14)**	**GLDM(14)**
Elongation	Energy	GLNU	Autocorrelation	GLNU	Dependence Entropy	SDE
Flatness	Total Energy	GLNUN	Cluster Prominence	GLNUN	DNU	LDE
Least Axis	Entropy	LRE	Cluster Tendency	HGLZE	DNUN	GLN
Major Axis	10Percentile	LRHGLE	Contrast	LAE	Dependence Variance	DN
Minor Axis	90Percentile	LRLGLE	Correlation	LAHGE	GLNU	DNN
M2DDC	Interquartile Range	LRLGLE	Difference Average	LALGE	GLV	GLV
M2DDR	Kurtosis	LGLRE	Difference Entropy	SZNU	HGLE	DV
M2DDS	Maximum	Run Entropy	Difference Variance	SZNUN	LDE	DE
M3DD	Minimum	RLNU	Id	SAE	LDLGLE	LGLE
Sphericity	Mean	RLNUN	Idm	SAHGE	LDHGLE	HGLE
SurfaceArea	MAD	Run Percentage	Idmn	SALGE	LGLE	SDLGLE
SVR	Median	Run Variance	Idn	Zone Entropy	SDE	SDHGLE
Volume	Range	SRE	Imc1	Zone Percentage	SDHGE	LDHGLE
Mesh Volume	rMAD	SRHGE	Imc2	Zone Variance	SDLGE	LDLGLE
	RMS	SRLGE	Inverse Variance	GLV		
	Standard Deviation	GLV	Joint Average	LGLZE		
	Uniformity		Joint Energy			
	Variance		Joint Entropy			
	Skewness		Max Probability			
			Sum Entropy			
			Sum Squares			

Abbreviations: GLRLM: Gray Level Run Length Matrix; GLCM: Gray Level Co-occurrence Matrix; GLSZM: Gray Level Size Zone Matrix; NGTDM: Neighboring Gray Tone Difference Matrix; GLDM: Gray Level Dependence Matrix.
